# Protein evolution on a human signaling network

**DOI:** 10.1186/1752-0509-3-21

**Published:** 2009-02-18

**Authors:** Qinghua Cui, Enrico O Purisima, Edwin Wang

**Affiliations:** 1 Biotechnology Research Institute, National Research Council Canada, Montreal, Quebec, Canada; 2 Center for Bioinformatics, McGill University, Montreal, Quebec, Canada; 3 Department of Medical Informatics, Peking University Health Science Center, Beijing, PR China

## Abstract

**Background:**

The architectural structure of cellular networks provides a framework for innovations as well as constraints for protein evolution. This issue has previously been studied extensively by analyzing protein interaction networks. However, it is unclear how signaling networks influence and constrain protein evolution and conversely, how protein evolution modifies and shapes the functional consequences of signaling networks. In this study, we constructed a human signaling network containing more than 1,600 nodes and 5,000 links through manual curation of signaling pathways, and analyzed the *d*_N_/*d*_S _values of human-mouse orthologues on the network.

**Results:**

We revealed that the protein *d*_N_/*d*_S _value decreases along the signal information flow from the extracellular space to nucleus. In the network, neighbor proteins tend to have similar *d*_N_/*d*_S _ratios, indicating neighbor proteins have similar evolutionary rates: co-fast or co-slow. However, different types of relationships (activating, inhibitory and neutral) between proteins have different effects on protein evolutionary rates, i.e., physically interacting protein pairs have the closest evolutionary rates. Furthermore, for directed shortest paths, the more distant two proteins are, the less chance they share similar evolutionary rates. However, such behavior was not observed for neutral shortest paths. Fast evolving signaling proteins have two modes of evolution: immunological proteins evolve more independently, while apoptotic proteins tend to form network components with other signaling proteins and share more similar evolutionary rates, possibly enhancing rapid information exchange between apoptotic and other signaling pathways.

**Conclusion:**

Major network constraints on protein evolution in protein interaction networks previously described have been found for signaling networks. We further uncovered how network characteristics affect the evolutionary and co-evolutionary behavior of proteins and how protein evolution can modify the existing functionalities of signaling networks. These new insights provide some general principles for understanding protein evolution in the context of signaling networks.

## Background

Proteins in cells tend to form a complex cellular signaling network that responds to various signals, ranging from environmental conditions, hormones or neurotransmitters to ions, and perform a series of tasks such as cell growth, maintenance of cell survival, proliferation, differentiation, development and apoptosis [1-4]. Cellular signaling networks are ubiquitous in various prokaryotes and eukaryotes and play pivotal roles in fundamental processes. Most studies on signaling have so far focused on certain particular signaling pathways or cascades, which represent a family of genes or specific biological processes. However, signaling pathways normally cross talk, branch out, form loops and are linked together to form a complex network. Therefore, it is necessary to study biological questions in a broader network context [5-7]. At present, one of the obstacles to performing large-scale analysis of signaling networks is the lack of a comprehensive signaling network dataset, because cellular signaling information is scattered in literature. So far only a few studies have been conducted for understanding topological organization, cancer signaling and microRNA regulation on literature-mined signaling networks [2,8-10].

At the molecular level, the architectural structure of cellular networks could provide constraints and functional innovations for protein evolution. Using protein interaction networks, previous studies addressing this question analyzed the conservation of network motifs [11,12], link numbers, interacting partners and functional modules of the network proteins [13-15] and regions of network topology [16]. Although cellular signaling is one of the most important biological processes, how signaling networks provide constraints on protein evolution and what functional consequences of signaling networks are caused by protein evolution have not been studied. To address these questions, we used our previously literature-mined human cellular signaling network which contains more than 1,600 nodes and 5,000 interactions [8,10] to systematically analyze the *d*_N_/*d*_S _of human-mouse orthologues on the human signaling network.

## Results

To understand how the architectural structure of signaling networks provides constraints for protein evolution, we first constructed a human signal transduction network by manually curating signaling pathways [8,10]. We merged the curated data with other literature-mined human cellular signaling pathways such as a small signaling network containing ~500 genes [2]. As a result, the signaling network contains ~1,600 nodes and ~5,000 interactions [10]. In the network, nodes represent proteins/genes, while neutral and directed links represent physical interactions and activating/inhibitory relations between proteins, respectively. Directed links have two types: positive links (an upstream protein activates a downstream protein) and negative links (an upstream protein inhibits a downstream protein). The network contains 2,403, 741, 1,915 and 30 links with positive, negative, neutral and unknown type, respectively. To study the evolutionary rate of the proteins in the network, we mapped the *d*_N_/*d*_S _values of human and mouse orthologues onto the network proteins. The value of *d*_N_/*d*_S _is the ratio of the rate of DNA substitutions affecting the amino-acid composition of the gene product (*d*_N_) to the rate of DNA substitutions that are silent at the amino-acid level (*d*_S_). The value of *d*_N_/*d*_S _can be used to measure the rate of protein evolution after controlling for mutation rate [17]. Therefore, in this study, we used *d*_N_/*d*_S _as a metric to measure the rate of protein evolution. The *d*_N_/*d*_S _values were calculated based on the *d*_N _and *d*_S _values which have been deposited in the database H-InvDB (see Methods).

### Protein evolutionary rates differ along the signaling information flow

Normally, cellular signaling information flow propagates from the extracellular space to the nucleus. Therefore, we asked how protein evolutionary rates vary along the signaling information flow. To answer this question, we first sorted the network proteins into four groups: extracellular space, membrane, intracellular space, and nucleus, based on their cellular locations in the signaling information flow. We then calculated the average *d*_N_/*d*_S _in each group. We found that the average *d*_N_/*d*_S _are different for each group along the signal information flow (Table 1). These results suggest that proteins in different stages of the signaling information flow (different cellular locations) evolve at different rates, and further indicate that different cellular compartments have different protein evolutionary rate. Proteins in extracellular space and cellular membrane account for the fastest evolving proteins, while proteins in intracellular space and nucleus account for the slowest evolving proteins. Proteins in these two groups show significantly different evolutionary rates (median *d*_N_/*d*_S_: 0.124 vs. 0.088, 2.5% and 97.5% percentage quantiles, [0.007, 0.668] and [0.000, 0.463], respectively, P = 3.36 × 10^-7^).

**Table 1:** Protein evolutionary rates distribution along the signaling information flow

**Cellular location**	**Extracellular**	**Membrane**	**Cytoplasm**	**Nucleus**
*d*_N_/*d*_S _(median)	0.220	0.108	0.086	0.094
P value*	1.36 × 10^-5^	0.01	0.04	0.13

*The P values were calculated by Wilcoxon test by comparing the protein evolutionary rates in each group with the evolutionary rates of the whole network proteins.

### Protein evolutionary rates are associated with network features

We performed a detailed analysis of the protein evolutionary rate on the network using several network features. Let Δ_ij_(*d*_N_/*d*_S_) represent the difference in *d*_N_/*d*_S _for a pair of genes. We calculated Δ_ij_(*d*_N_/*d*_S_) for all the pairs of genes (network nodes), which are connected by either a directed or neutral link. Directed links are signaling interactions that activate or inhibit while neutral links are just physical interactions. We also did the same for an equal number of random gene pairs in the network. We found that Δ_ij_(*d*_N_/*d*_S_) is significantly smaller for connected pairs of genes than that of random pairs (median value 0.072 vs. 0.092, 2.5% and 97.5% percentage quantiles, [0.002, 0.506] and [0.004, 0.536], respectively, P = 5.66 × 10^-13^, Wilcoxon Test). This result indicates that interacting proteins in the network tend to evolve together: co-fast or co-slow. In signaling networks, proteins have different relationships. We asked whether different types of interactions have different constraints on protein evolution in the network. To address this question, we classified the links into three groups according to their link types: neutral link, positive (activating) link, and negative (inhibiting) link groups. We calculated the average Δ_ij_(*d*_N_/*d*_S_) for protein pairs in each group, respectively. We found that these three types of links act differently on protein co-evolution. The median values for neutral, positive and negative link groups are: 0.067, 0.072, and 0.088, respectively. The 2.5% and 97.5% percentage quantiles are [0.003, 0.359], [0.002, 0.601] and [0.003, 0.507], respectively (P = 0.02, Kruskal-Wallis test). Negative links account for the highest median Δ_ij_(*d*_N_/*d*_S_), followed by the positive links and neutral links (P = 0.02, P = 0.003, respectively). It is also clear that the signaling link group (combining the positive and negative link groups) has a higher median Δ_ij_(*d*_N_/*d*_S_) value than the neutral link group (median value 0.075 vs. 0.067, 2.5% and 97.5% percentage quantiles, [0.002, 0.601] and [0.003, 0.359], respectively, P = 0.04, Wilcoxon test). These results hint that the types of interactions between proteins in the network can have different effects on the co-evolution of the proteins. Neutral links representing protein-protein interactions within protein complexes in the signaling network tend to have more similar evolutionary rates and might be more co-evolved. Physically interacting proteins in signaling networks often form protein complexes that are used for isolating certain signaling cascades from other reactions, or for signaling protein translocations. Therefore, co-evolution of the physically interacting proteins could enhance the coordination of these processes. Positive and negative links represent the reactions exerted by signaling enzymes, i.e., kinases and phosphatases. Unlike neutral links, these appear to co-evolve less and might have different evolutionary mechanisms (see more in Discussion).

**Figure 1:**
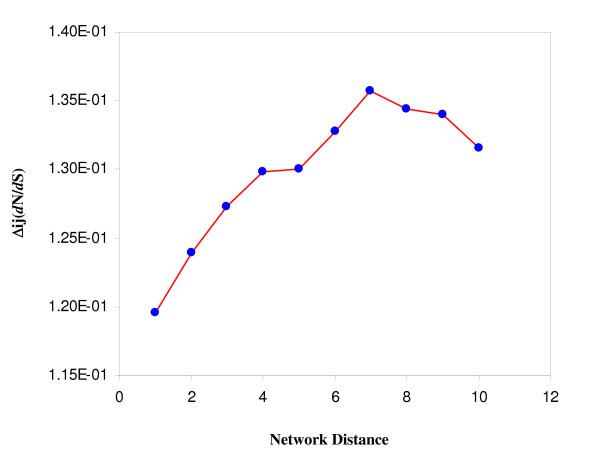
**Correlation between network distance and Δ_ij_(*d*_N_/*d*_S_)**. Δ_ij_(*d*_N_/*d*_S_), the absolute difference of *d*_N_/*d*_S _was calculated for all pairs of genes, and plotted against the network distance, defined by the shortest directed path between them.

To further differentiate the evolutionary behavior of directed and neutral links, we investigated the association of the distance between two proteins in the network with their evolution rate. In the network, signals can be transduced from one node to another through many different cascades, one of which contains the least number of links and is called the shortest path. We defined the network distance to be the shortest path between two nodes. We first sorted the shortest paths between any two nodes in the network using Dijkstra's algorithm. The shortest paths consisting of directed and neutral links were examined independently. We calculated the Δ_ij_(*d*_N_/*d*_S_) between all pairs of nodes having either a directed or neutral shortest path. For either path type, we grouped the pairs of proteins according to the length of their shortest paths (network distances) and calculated the average Δ_ij_(*d*_N_/*d*_S_) in each group. As shown in Figure 1, Δ_ij_(*d*_N_/*d*_S_) increases as the network distance increases when the directed shortest path was examined (Spearman's correlation R = 0.83, P = 0.005). These results indicate that for the directed shortest paths, the more distant two proteins are, the less chance they share similar evolutionary rates. The same could not be said with statistical confidence for the neutral shortest path (Spearman's correlation R = 0.61, P = 0.063). However, as shown in Figure 1, when the length of their shortest paths is greater than 7, the average Δ_ij_(*d*_N_/*d*_S_) starts to decrease. To understand this observation, we defined the types of node pairs and calculated the fractions of node-pair-types for each group. We defined node-pair-types based on the cellular locations (i.e., extracellular space, membrane, intracellular space, and nucleus) of the nodes in each pair [see Additional file 1], i.e., for a node pair, if one node is located in intracellular space and the other is located in nucleus, we defined this pair as a Cy (intracellular space)-Nu (nucleus) type. As shown in Additional file 1, the group average Δ_ij_(*d*_N_/*d*_S_) is affected by the fractions of the node-pair-types. In particular, two types of the node pairs in each group, Cy-Cy and Ex-Cy might play important roles for the group average Δ_ij_(*d*_N_/*d*_S_) [see Additional file 1].

As we uncovered above, interacting proteins in the network show similar evolutionary rates. To understand this phenomenon in more detail quantitatively, we extracted the network components in which the proteins have similar *d*_N_/*d*_S_. A network component is a connected sub-network. Two nodes are in a same network component if there is a path between them. We identified network components formed by proteins with *d*_N_/*d*_S _values in the top 10% (*d*_N_/*d*_S _> 0.316) and the bottom 10% (*d*_N_/*d*_S _< 0.016), respectively. We found that both groups of proteins tend to form bigger network components than a randomly selected of proteins consisting of 10% of the network nodes (P = 0.004 and 0.0002, respectively, randomization tests).

To understand the functional consequences of the low and fast evolving network proteins, we analyzed the enrichment of biological functions of the proteins in the highest and lowest 10% of *d*_N_/*d*_S _proteins, respectively, using FatiGO software tool [18]. The analysis revealed that high *d*_N_/*d*_S _proteins are significantly enriched with apoptotic signaling (P = 8.9 × 10^-7^) and immunological signaling (P = 9.6 × 10^-6^), while low *d*_N_/*d*_S _proteins (*d*_N_/*d*_S _< 0.016) are significantly enriched with GTP binding (P = 1.0 × 10^-7^) and hydrolase activity (P = 3.3 × 10^-6^). Because a higher *d*_N_/*d*_S _value represents fast evolution of a protein, these results suggest that the proteins of apoptotic signaling and immunological signaling are highly divergent. Although both apoptotic and immunological signaling are intensively involved in host defense responses, they evolve in different ways. More specifically, among proteins in the highest 10% *d*_N_/*d*_S_, apoptotic signaling proteins preferentially form network components with other proteins, i.e., 18 out of 28 proteins in the largest network component (which we called it 28-cluster) are signaling proteins. In contrast, immunological signaling proteins (antigens) in the same top 10%*d*_N_/*d*_S _group, were isolated and were not part of large network components. Independently fast-evolving antigens will increase the diverse responses of the host cells. On the other hand, interdependently fast-evolving apoptotic signaling proteins (i.e., the 28-cluster) might enhance coordinated responses from the host cells and the rapid information transfer needed for survival of the organisms.

We further catalogued the orthologues of the 28-cluster proteins across several model organisms such as *Escherichia coli*, yeast (*Saccharomyces cerevisiae*), worm (*Caenorhabditis elegans*), fly (*Drosophila melanogaster*) and zebrafish (*Danio rerio*). A similar analysis was also extended to whole network genes. Not surprisingly, the 28-cluster proteins have much fewer orthologues in the model organisms than the network proteins (Table 2). These results indicate that high *d*_N_/*d*_S _apoptotic signaling proteins (*d*_N_/*d*_S _> 0.316) lead to multiple and more flexible and adaptive cell death signaling pathways in human. Indeed, only one primitive dedicated apoptotic signaling pathway is known in *C. elegans *[19], while several cell death signaling pathways have evolved in human and mouse genomes. Extensive expansion of apoptotic signaling proteins in human leads to the integration of a significant portion of apoptotic proteins into the signaling processes that are used in normal physiological conditions. For example, apoptotic proteins such as caspases are involved in many non-apototic signaling processes in human and mouse, i.e., cell proliferation and differentiation [20,21]. In mice, caspase-9 is involved in both apoptosis and inner ear epithelium development [22], while caspase-8 is involved in critical signaling for cardiac and neural development during early embryogenesis [23]. Conversely, multiple normal signaling mechanisms have been recruited to cell death either as backups or parallel mechanisms of apoptosis. For example, cytochrome c is a key electron carrier of mitochondrial complex III for respiration. However, in mammals cytochrome c is involved in apoptosis when mitochondria are damaged [24]. As a result, the mammalian cell death machinery is intertwined with multiple cellular signaling processes that are part of normal cellular physiological signaling processes, providing backups and flexible signaling mechanisms to cell death signaling. We found that ten out of the 28-cluster proteins are not apoptotic proteins. Fast co-evolution of apoptotic proteins with other proteins would enhance the rapid information transfer between apoptotic signaling pathways and other pathways. These diverse and flexible apoptotic signaling makes possible a rapid response to a variety of complex internal and external stress signals. Finally, the co-evolution of network components significantly promotes new functionalities arising from the integration of diverse signaling cascades in signaling networks.

**Table 2:** Percentage of orthologues of the human signaling network proteins across species

	***E. coli***	***S. cerevisiae***	***C. elagans***	***D. melanogaster***	***D. rerio***
28-cluster protein	0	0	0	25%	64%
Network protein	5%	11%	26%	61%	68%

### Sensitivity analysis

The human signaling network is incomplete and contains errors. In order to investigate the potential effects of data incompleteness and possible errors, we performed a sensitivity analysis by randomly removing 10% of the links and adding the same number of random links into the network. By doing so, we have artificially introduced approximately 10% false negatives and 10% false positives into the network. We examined the effect on the main results described in the previous sections. For protein co-evolution, we found that Δ_ij_(*d*_N_/*d*_S_) is still significantly less than for a random pair (median value 0.074 vs. 0.081, 2.5% and 97.5% percentage quantiles, [0.002, 0.507] and [0.004, 0.580], respectively, P = 2.5 × 10^-9^, Wilcoxon Test). We found that the three types of links still contribute differently to protein co-evolution (median: 0.069, 0.076, 0.084, 2.5% and 97.5% percentage quantiles, [0.003, 0.384], [0.002, 0.586] and [0.004, 0.495], respectively, P = 0.09, Kruskal-Wallis test). As before, negative links account for the highest Δ_ij_(*d*_N_/*d*_S_), followed by the positive links and neutral links (P = 0.06, P = 0.01, respectively). It is also clear that the signal link group (combining the positive link and negative link groups) has higher Δ_ij_(*d*_N_/*d*_S_) than the neutral link group (median value 0.078 vs. 0.069, 2.5% and 97.5% percentage quantiles, [0.002, 0.573] and [0.003, 0.384], respectively, P = 0.06, Wilcoxon test). The Δ_ij_(*d*_N_/*d*_S_) increases as the network distance increases (Spearman's correlation R = 0.636, P = 0.05 for the directed path). We found that proteins belonging to network components with the highest and lowest 10% *d*_N_/*d*_S _values still tend to form bigger network components than a randomly selected set of 10% of the proteins in the network (P = 0.004 and 0.0002, respectively, randomization tests). These results indicate that most of the major conclusions in this study remain unchanged by the addition of a moderate amount of false positives and false negatives. Therefore, the results we obtained are fairly robust.

## Discussion

Previous studies in protein interaction network evolution have made several major conclusions: (a) hub proteins or proteins having more interacting links tend to be more conserved [25]; (b) proteins in the network periphery undergo positive selection while those in the network center are more conserved [16]; (c) network proteins appear to be co-evolved with their neighbors [25]; (d) interacting proteins with high local clustering tend to be more conserved [26].

In this study, we constructed a human signaling network and analyzed the protein evolutionary rate on the network. Consistent with the studies of protein interaction networks, we find that proteins appear to be co-evolved with their neighbors in the signaling network. However, in our analysis, we further found that in signaling networks different types of interactions have different strength of constraints on protein co-evolution, in which proteins linked by physical interactions tend to be more co-evolved. Furthermore, for directed shortest paths, the more distant two proteins have, the less chance they share similar evolutionary rates. However, such a correlation was not observed with respect to the neutral shortest path. Positive and negative links in signaling networks include the major signaling regulatory mechanism: protein phosphorylation and dephosphorylation, which are exerted by kinases and phosphatases. Both types of signaling enzymes are multiple domain proteins which often contain, in addition to their core catalytic function, multiple independently folding domains or motifs that mediate connectivity by interacting with other signaling elements [27]. Therefore, signaling enzymes are known to have high modular strategies for controlling their input and output connectivities: the core catalytic activity of a signaling protein is physically and functionally separable from molecular domains or motifs that determine its linkage to both inputs and outputs. These features of signaling enzymes suggest that they have distinct evolutionary mechanisms from other proteins, i.e., insertion and recombination of modules are suggested to be a common mechanism of the evolution of new proteins and connections [27,28]. Collectively, these features of signaling enzymes might explain the evolutionary rates differences between the signaling enzymes and their connecting partners. Furthermore, negative regulators such as phosphatases are more promiscuous in their selectivity for their targets/substrates. This fact might explain why phosphatases (forming negative links in the network) have even weaker co-evolution rates with their connecting partners. On the other hand, neutral links represent physical protein interactions in the signaling network. Physically interacting proteins in signaling networks often form protein complexes that are used for isolating certain signaling cascades from other reactions, or signaling protein translocations. Therefore, co-evolution of the physically interacting proteins will enhance the coordination of the processes mentioned above.

In this study we showed that extracellular proteins are evolving faster, which is in agreement with several previous studies [16,29]. Signaling proteins in the extracellular space are the stimuli of intra- and inter-cell signaling. Fast evolving proteins in the extracellular space allow cells to explore various responses to new stimuli and might establish novel communications between cells. This would promote the cell's capability to respond and adapt to environmental changes and explore new environmental niches. Recently, Kim et al. showed that proteins in the peripheral regions (i.e., extracellular and membrane proteins) of protein interaction networks undergo positive selection, while proteins in the center of the protein interaction networks are conserved [16]. Protein interaction networks collect the global protein interactions in the cell while signaling networks represent a part of cell activities (i.e., cell signaling) [3]. The extracellular components of the signaling network are similar to the peripheral regions of protein interaction and gene regulatory networks, which count for many adaptive properties of the organism [16,30]. Consistently, both Kim et al. [16] and our studies show that proteins in this region are fast evolving. In this study, we further showed that evolutionary rates of proteins decrease along the signaling information flow from extracellular space (input layer), intracellular space to nucleus (output layer). The downstream portion of the signaling network evolves more conservatively. This is understandable given that the downstream segment of the signaling network ultimately governs cellular behavior and activities. It is therefore not surprising to find that tumor driver mutating genes, even highly mutated ones, are enriched in the downstream portion of human signaling network [8,10]. The existence of fast and slowly evolving proteins in the signaling network upstream and downstream portions, respectively, suggests that proteins in the upstream portion of the signaling flow are more adaptable and could be more easily rewired to generate different combinatory regulation mechanisms for the downstream portion of the signaling flows. Thus, it would be more critical to regulate the genes in the downstream portion of the network. Indeed, we do find that the genes in the downstream portion of the signaling network are more significantly regulated by microRNAs than the upper portion of the signaling information flow [9].

It is known that apoptotic and immunological signaling proteins are fast evolving. However, using a network approach, we found that both signaling processes have different modes of evolution: fast evolving immunological signaling proteins are more independent, while fast evolving apoptotic signaling proteins tend to form network components and co-evolve with other signaling proteins. Apoptotic signaling proteins are extensively expanded in mammalian genomes in comparison to other genomes such as those of yeast and fly. The diverse and flexible apoptotic signaling makes it possible for mammals to rapidly respond to a variety of complex internal and external stress signals. Finally, the functional consequences of co-evolution of the apoptotic proteins by forming network components significantly enhance the integration of diverse signaling cascades to cell death signaling and make the information transfer more efficient between apoptotic signaling and other signaling pathways. Our findings will improve our understanding of signaling protein evolution and the mechanism of signal integration in signaling networks caused by protein evolution.

## Conclusion

Several major conclusions on protein evolution in protein interaction networks have been previously described. In this work, we further uncovered how network characteristics affect the evolutionary and co-evolutionary behavior of proteins. For example, we showed that in signaling networks different types of interactions have different strength of constraints on protein co-evolution, in which proteins linked by physical interactions tend to be more co-evolved. Furthermore, for directed shortest paths, the more distant two proteins have, the less chance they share similar evolutionary rates. However, such a correlation was not observed with respect to the neutral shortest path. We further showed that evolutionary rates of proteins decrease along the signaling information flow from extracellular space (input layer), intracellular space to nucleus (output layer). The downstream portion of the signaling network evolves more conservatively.

Our analysis further suggested how protein evolution could modify the existing functionalities of signaling networks. For example, we showed that fast evolving apoptotic signaling proteins tend to form network components and co-evolve with other signaling proteins. The diverse and flexible apoptotic signaling makes it possible for mammals to rapidly respond to a variety of complex internal and external stress signals. Finally, the functional consequences of co-evolution of the apoptotic proteins by forming network components significantly enhance the integration of diverse signaling cascades to cell death signaling and make the information transfer more efficient between apoptotic signaling and other signaling pathways. These new insights provide some general principles for understanding protein evolution in the context of signaling networks.

## Methods

### Datasets

The human-mouse protein *d*_N _and *d*_S _data were downloaded from H-InvDB . We calculated the *d*_N_/*d*_S _value for each protein [see Additional file 2]. We extracted human-mouse orthologues from a database, Inparanoid (hsamus_ortholog.txt, ).

### Signaling network construction

To construct the human cellular signaling network, we manually curated signaling pathways from the BioCarta database , which so far is the most comprehensive database for cellular signaling pathways. The curated pathway dataset recorded gene names and functions, cellular locations of each gene and relationships between the genes. We merged these genes and their interactions with another literature-mined signaling network that contains ~500 proteins [2]. To ensure the accuracy and the consistency of the data, each referenced pathway was cross-checked by different researchers and finally all the documented pathways were checked by one researcher. As a result, the merged signaling network contains more than 1,600 nodes and 5, 000 links [8,10]. The human signaling network data are accessible from Cui et al [10].

### Gene Ontology analysis

To examine the enrichment of biological processes for a set of genes, we used FatiGO tool [18] and the default parameters. The whole network genes were used as a background gene set.

### Statistical analysis

We performed Wilcoxon tests, Kruskal-Wallis tests, and Spearman's correlation using R, a software environment for statistical computing . Details for randomization tests of cellular networks have been described previously [31]. Briefly, randomization tests of the network components formed by a set of genes were conducted by taking the same number of genes randomly from the network for 5,000 times and calculating its network components each time.

## Authors' contributions

QC carried out the analysis, EW conceived of the study, and participated in its design and coordination. QC, EW and EOP wrote the manuscript. All authors read and approved the final manuscript.

## Supplementary Material

Additional file 1 **Fractions of node-pair-types in each shortest path group.** The data provided represent the analysis of fractions of the node-pair-types in each shortest path group. Click here for file

Additional file 2 **The values of *d*_N_/*d*_S _of the human-mouse orthologues on the human signaling network.** The file provides the *d*_N_/*d*_S _data for the human-mouse orthologues on the human signaling network. Click here for file

## References

[B1] Balazsi G, Barabasi AL, Oltvai ZN (2005). Topological units of environmental signal processing in the transcriptional regulatory network of Escherichia coli. Proc Natl Acad Sci USA.

[B2] Ma'ayan A, Jenkins SL, Neves S, Hasseldine A, Grace E, Dubin-Thaler B, Eungdamrong NJ, Weng G, Ram PT, Rice JJ (2005). Formation of regulatory patterns during signal propagation in a Mammalian cellular network. Science.

[B3] Wang E, Lenferink A, O'connor-McCourt M (2007). Cancer systems biology: exploring cancer-associated genes on cellular networks. Cell Mol Life Sci.

[B4] Yarden Y, Sliwkowski MX (2001). Untangling the ErbB signalling network. Nat Rev Mol Cell Biol.

[B5] Vidal M (2005). Interactome modeling. FEBS Lett.

[B6] Letunic I, Yamada T, Kanehisa M, Bork P (2008). iPath: interactive exploration of biochemical pathways and networks. Trends Biochem Sci.

[B7] Karimpour-Fard A, Leach SM, Hunter LE, Gill RT (2008). The topology of the bacterial co-conserved protein network and its implications for predicting protein function. BMC Genomics.

[B8] Awan A, Bari H, Yan F, Mokin S, Yang S, Chowdhury Q, Yu Z, Purisima EO, Wang E (2007). Regulatory network motifs and hotspots of cancer genes in a mammalian cellular signaling network. IET Syst Biol.

[B9] Cui Q, Yu Z, Purisima EO, Wang E (2006). Principles of microRNA regulation of a human cellular signaling network. Mol Syst Biol.

[B10] Cui Q, Ma Y, Jaramillo M, Bari H, Awan A, Yang S, Zhang S, Liu L, Lu M, O'connor-McCourt M (2007). A map of human cancer signaling. Mol Syst Biol.

[B11] Wuchty S, Oltvai ZN, Barabasi AL (2003). Evolutionary conservation of motif constituents in the yeast protein interaction network. Nat Genet.

[B12] Mazurie A, Bottani S, Vergassola M (2005). An evolutionary and functional assessment of regulatory network motifs. Genome Biol.

[B13] Amoutzias GD, Pichler EE, Mian N, De GD, Imsiridou A, Robinson-Rechavi M, Bornberg-Bauer E, Robertson DL, Oliver SG (2007). A protein interaction atlas for the nuclear receptors: properties and quality of a hub-based dimerisation network. BMC Syst Biol.

[B14] Beltrao P, Serrano L (2007). Specificity and evolvability in eukaryotic protein interaction networks. PLoS Comput Biol.

[B15] Berg J, Lassig M, Wagner A (2004). Structure and evolution of protein interaction networks: a statistical model for link dynamics and gene duplications. BMC Evol Biol.

[B16] Kim PM, Korbel JO, Gerstein MB (2007). Positive selection at the protein network periphery: evaluation in terms of structural constraints and cellular context. Proc Natl Acad Sci USA.

[B17] Swanson WJ, Vacquier VD (2002). The rapid evolution of reproductive proteins. Nat Rev Genet.

[B18] Al-Shahrour F, Minguez P, Tarraga J, Medina I, Alloza E, Montaner D, Dopazo J (2007). FatiGO +: a functional profiling tool for genomic data. Integration of functional annotation, regulatory motifs and interaction data with microarray experiments. Nucleic Acids Res.

[B19] Metzstein MM, Stanfield GM, Horvitz HR (1998). Genetics of programmed cell death in C. elegans: past, present and future. Trends Genet.

[B20] Kuida K, Haydar TF, Kuan CY, Gu Y, Taya C, Karasuyama H, Su MS, Rakic P, Flavell RA (1998). Reduced apoptosis and cytochrome c-mediated caspase activation in mice lacking caspase 9. Cell.

[B21] Yoshida H, Kong YY, Yoshida R, Elia AJ, Hakem A, Hakem R, Penninger JM, Mak TW (1998). Apaf1 is required for mitochondrial pathways of apoptosis and brain development. Cell.

[B22] Cecconi F, Roth KA, Dolgov O, Munarriz E, Anokhin K, Gruss P, Salminen M (2004). Apaf1-dependent programmed cell death is required for inner ear morphogenesis and growth. Development.

[B23] Sakamaki K, Inoue T, Asano M, Sudo K, Kazama H, Sakagami J, Sakata S, Ozaki M, Nakamura S, Toyokuni S (2002). Ex vivo whole-embryo culture of caspase-8-deficient embryos normalize their aberrant phenotypes in the developing neural tube and heart. Cell Death Differ.

[B24] Liu X, Kim CN, Yang J, Jemmerson R, Wang X (1996). Induction of apoptotic program in cell-free extracts: requirement for dATP and cytochrome c. Cell.

[B25] Stumpf MP, Kelly WP, Thorne T, Wiuf C (2007). Evolution at the system level: the natural history of protein interaction networks. Trends Ecol Evol.

[B26] Wuchty S, Barabasi AL, Ferdig MT (2006). Stable evolutionary signal in a yeast protein interaction network. BMC Evol Biol.

[B27] Bhattacharyya RP, Remenyi A, Yeh BJ, Lim WA (2006). Domains, motifs, and scaffolds: the role of modular interactions in the evolution and wiring of cell signaling circuits. Annu Rev Biochem.

[B28] Lander ES, Linton LM, Birren B, Nusbaum C, Zody MC, Baldwin J, Devon K, Dewar K, Doyle M, FitzHugh W (2001). Initial sequencing and analysis of the human genome. Nature.

[B29] Waterhouse RM, Kriventseva EV, Meister S, Xi Z, Alvarez KS, Bartholomay LC, Barillas-Mury C, Bian G, Blandin S, Christensen BM (2007). Evolutionary dynamics of immune-related genes and pathways in disease-vector mosquitoes. Science.

[B30] Davidson EH, Erwin DH (2006). Gene regulatory networks and the evolution of animal body plans. Science.

[B31] Wang E, Purisima E (2005). Network motifs are enriched with transcription factors whose transcripts have short half-lives. Trends Genet.

